# Healing Oral Mucositis With *Arrabidaea chica* Verlot Mucoadhesive Gel in Patients Undergoing Hematopoietic Stem Cell Transplantation: A Randomized Controlled Clinical Trial

**DOI:** 10.1155/bmri/3700170

**Published:** 2025-12-12

**Authors:** Núbia de Cássia Almeida Queiroz, Ilza Maria de Oliveira Sousa, Rogério José Machado Júnior, Afonso Celso Vigorito, Elida Adriana de Castro Aranha, Andressa Gomes Melo, João Ernesto de Carvalho, Mary Ann Foglio

**Affiliations:** ^1^ Faculty of Medical Sciences, State University of Campinas, Sao Paulo, Brazil, unicamp.br; ^2^ Faculty of Pharmaceutical Sciences, State University of Campinas, Sao Paulo, Brazil, unicamp.br

**Keywords:** *Arrabidaea chica*, *Fridericia chica*, healing, oral mucositis

## Abstract

**Introduction:**

Oral mucositis affects up to 80% of patients undergoing bone marrow transplantation, causing painful ulcers, increased infection risk, and impaired quality of life. Current treatments are mainly palliative, highlighting the need for new therapeutic options. *Arrabidaea chica* Verlot extract has shown wound healing potential through antioxidant activity and collagen synthesis promotion in vitro and significant wound area reduction in vivo.

**Objective:**

The objective of the study is to evaluate the wound healing potential of a mucoadhesive gel containing 2.5% *A. chica* extract for treating oral mucositis in oncohematological patients undergoing bone marrow transplantation.

**Methods:**

This was a randomized, controlled, open‐label, parallel‐group clinical trial conducted at HC/UNICAMP from October 2022 to November 2024. Forty‐four patients were randomized to the intervention group (mucoadhesive *A. chica* gel, *n* = 22) or control group (low‐intensity laser therapy, *n* = 22). The primary outcome was the time to healing of oral mucositis. Statistical analysis compared mean healing times between groups.

**Results:**

Twenty participants in the intervention group and 18 in the control group completed the study. Mean healing time was 5.2 ± 1.5 days for the intervention group and 11.2 ± 6.5 days for the control group, representing a 2.2‐fold faster healing with *A. chica* gel. Some intervention participants reported a mild burning sensation, without affecting treatment adherence or efficacy.

**Conclusion:**

The mucoadhesive gel containing *A. chica* extract proved to be a safe and effective therapeutic option for treating oral mucositis in patients undergoing bone marrow transplantation.

**Trial Registration:**

ReBEC number: RBR‐5×4397

## 1. Introduction

Cancer remains a major global public health challenge. In 2022, approximately 20 million new cases were diagnosed worldwide. Standard treatment modalities, including chemotherapy, radiotherapy, or their combination, are associated with significant adverse effects, among which oral mucositis (OM) is particularly frequent and debilitating [1, 2].

OM is a painful, ulcerative inflammatory condition of the oral mucosa that can compromise patients′ ability to tolerate cancer treatments, with incidence ranging from 15% in low‐risk therapies to 60%–100% in high‐dose chemotherapy, radiotherapy, or bone marrow transplantation. OM is associated with secondary infections, adverse clinical outcomes, and increased healthcare costs, and severe cases may require dose reductions or early discontinuation of treatment, negatively affecting efficacy, survival, and quality of life. Current management focuses on symptom relief and complication prevention, including anti‐inflammatory and antimicrobial agents, mucoprotective substances, nutritional support, growth factors, cryotherapy, herbal medicines, and low‐intensity laser therapy. MASCC/ISOO guidelines recognize low‐intensity laser therapy as a safe intervention for prophylaxis and treatment, reducing pain, lesion severity, and symptom duration; however, limited evidence supports its widespread clinical use. Given the absence of well‐established protocols for OM healing, there is a need to investigate new pharmacological agents, including natural products with therapeutic potential, as alternatives for prevention or lesion reversal [3–8].

In Brazil, *Arrabidaea chica* (Humb. & Bonpl.) Verlot is included in the National List of Medicinal Plants of Interest to the Unified Health System (Renisus). The plant′s pharmacological activity is largely attributed to its high content of polyphenols, especially flavonoids, with emphasis on anthocyanin derivatives such as 3‐deoxyanthocyanins. Key compounds include carajurina and carajurona, along with minor constituents like 3 ^′^‐hydroxycarajurona, 3 ^′^‐hydroxycarajurina, 6,7,3 ^′^,4 ^′^‐tetrahydroxy‐5‐methoxyflavilium, and 6,7,4 ^′^‐trihydroxy‐5‐methoxyflavilium. Extracts and fractions from the leaves of *A. chica* have demonstrated antimicrobial, anti‐inflammatory, antioxidant activity, and the ability to promote skin and tendon wound healing [9–11].

In vitro studies with *A. chica* leaf extract demonstrated stimulation of fibroblast proliferation, collagen synthesis, and antioxidant capacity. In vivo wound healing assays on the dorsal skin of rats showed accelerated and efficient lesion closure, achieving over 80% reduction in wound area. Formulations of the extract in hyaluronic acid micro‐ and nanoparticles further enhanced angiogenesis and tissue repair, likely through fibroblast activation and induction of angiogenesis during the proliferative phase of healing [11–14].

Phase 1 of the clinical study evaluated the safety of a mucoadhesive gel containing 2.5% *A. chica* extract in 20 healthy participants over 30 days, with application three times daily to four regions of the oral mucosa. No adverse events related to the gel were observed during weekly clinical assessments, diary records, or laboratory tests, and all participants completed the protocol. These findings, together with the extract′s wound healing potential, establish parameters for safety, efficacy, and reproducibility, supporting progression to Phase 2 and highlighting the value of natural products in managing symptoms associated with oncological treatments [15].

Considering the promising preliminary results, we conducted a Phase II/III prospective study to investigate the efficacy and safety of the mucoadhesive gel of *A. chica* Verlot containing 2.5% of the standardized extract to treat OM in patients with oncohematological diseases undergoing autologous and allogeneic bone marrow transplants (BMTs).

## 2. Methods

### 2.1. Study Design and Setting

This randomized, controlled clinical trial was conducted at the BMT Unit of the Hospital de Clínicas, State University of Campinas (UNICAMP), Campinas, Brazil, between October 2022 and November 2024.

### 2.2. Patient and Public Involvement

Patients and the public were not directly involved in the design, conduct, or reporting of this clinical trial. The protocol was developed based on clinical demands observed in routine care, with a focus on the management of OM in patients with oncohematological diseases.

### 2.3. Trial Design

This is a randomized, controlled, parallel‐group clinical trial designed to evaluate the wound healing efficacy of *A. chica* gel in patients with oncohematological diseases presenting OM. Participants were allocated to either the intervention group (*A. chica* gel) or the control group (CG) (low‐level laser therapy) in a 1:1 ratio using simple randomization, ensuring that each participant had an equal and independent chance of being assigned to either group. The study was conducted in a multicenter, open‐label design, with blinded assessment of clinical outcomes to minimize detection bias.

### 2.4. Changes to Trial Protocol

One of the protocol modifications in this trial was the exclusion of the photographic analysis of oral lesions using ImageJ software, which had been originally planned. This decision was made due to the inability to obtain standardized images, resulting from the anatomical variability and variable location of the lesions within the oral cavity, as well as the difficulty some participants experienced in keeping their mouths open during image capture because of pain, inflammation, and the extent of ulcerations.

In addition, the study population profile was modified. The original protocol included patients with head and neck cancer but was later adjusted to include oncohematological patients undergoing hematopoietic stem cell transplantation (HSCT). This change was implemented because these patients remain hospitalized throughout treatment, enabling more rigorous daily monitoring of both the intervention group and the CG during the management of OM. In the original population, home use of the gel presented challenges in ensuring adherence, while the CG received laser therapy only on the days of radiotherapy sessions at the hospital, with no treatment provided on weekends or public holidays. These inconsistencies in treatment compromised the ability to perform valid comparisons between the groups.

Finally, an additional research center was incorporated into the study to increase the sample size and diversify the patient population, thus establishing a multicenter trial.

#### 2.4.1. Inclusion Criteria

Inclusion criteria include the following:

• Both sexes
• Age above 18 years
• Participants with a previous diagnosis of oncohematological disease who underwent BMT
• Participants undergoing treatment in the unit who developed OM as a result of oncological treatment

#### 2.4.2. Exclusion Criteria

Exclusion criteria include the following:

• Participants with ulcerative lesions or infections in the oral cavity before chemotherapy treatment
• Participants using therapies other than those proposed by the study

### 2.5. Interventions

All patients in the BMT ward at the Hospital de Clínicas of Unicamp followed a protocol developed specifically to prevent OM, which includes the use of low‐intensity laser starting on the first day of the conditioning regimen (nonmyeloablative conditioning [NMA], reduced‐intensity conditioning [RIC], or myeloablative conditioning [MAC]), continuing until the engraftment of the marrow. The procedure was performed by a nurse from the BMT unit who had been previously trained.

The measurement of the severity of OM was performed by the medical and nursing team in the BMT ward using the World Health Organization (WHO) classification scale, defined as follows: (0) no abnormality detected; (1) presence of erythema, no specific treatment required; (2) painful condition, no need for analgesics, difficulty in eating; (3) presence of painful ulceration, requires analgesics, inability to eat; (4) presence of necrosis, parenteral feeding required.

When lesions of Grade I or higher developed despite preventive treatment, participants who consented to take part in the study were randomized into two groups: the test group (TG) and the CG. The TG was treated with a 2.5% *A. chica* mucoadhesive gel (Table 1), provided in individual sachets containing 2 g of the product. Participants were instructed to apply the gel directly to the lesion site three times a day, after main meals and oral hygiene. To optimize adhesion of the gel to the oral mucosa, participants were advised to avoid eating or drinking for 20 min following application. The first application was carried out under the supervision of the researcher, who provided detailed instructions on the application sites and proper use of the product.

**Table 1:** Composition of the 2.5% *A. chica* mucoadhesive gel.

**Ingredients**	**Concentration (%)**
Water	qs^a^ for 100
Glycerin	15.0
Hydroxyethylcellulose	8.5
Sodium benzoate	0.5
Potassium sorbate	0.5
Hyaluronic acid	0.01
L‐glutamine	2.0
Xylitol	1.0
*Arrabidaea chica* extract	2.5

^a^A sufficient quantity to complete (qs for) 100%.

The CG, which was treated with low‐level laser therapy, used the following parameters: red wavelength of 660 nm, fluence of 2 J/cm^2^, power ranging from 10 to 60 mW, and a total energy of 2 J per point, with an application time of 20 s per point. The therapy was performed concurrently with an infrared wavelength of 808 nm. The device model used was the Therapy EC by DMC ABC equipment.

### 2.6. Outcomes

The primary outcome was evaluated by the average duration of OM in days, from the onset of lesion treatment to complete healing, expressed as the mean and standard deviation (SD) for each group.

The secondary outcome included the evaluation of pain intensity using the Visual Analog Scale (VAS) and the assessment of health‐related quality of life through the European Quality of Life 5‐Dimensions (EQ‐5D) questionnaire.

### 2.7. Safety

All participants received a medication diary to record the application times and were instructed to report any side effects of the medication. Furthermore, the researcher′s phone number was available to all participants.

### 2.8. Sample Size

The sample size was calculated for the comparison of two independent means, with the primary outcome being the number of days for OM healing. The formula used was

n=2·Zα/2+Zβ2·σ2Δ2

where *α* = 0.05, study power = 80*%*, *σ* ^2^ represents the pooled variance, and *Δ* is the minimum clinically relevant difference, based on data from a pilot study. The initial calculation indicated 63 participants per group, to which 20% was added to account for potential dropouts, resulting in a total of 76 participants per group,

### 2.9. Sequence Generation

Block randomization of four participants was performed to ensure balance between the groups during recruitment. The randomization sequence was generated using the website http://www.randomization.com and was kept in sequentially numbered, opaque, sealed envelopes to conceal the allocation until interventions were assigned. The personnel responsible for enrolling and assigning participants to the interventions did not have access to the random allocation sequence.

### 2.10. Ethical Issues

The study was conducted in accordance with the ethical principles of the Declaration of Helsinki and the Brazilian National Health Council Resolution 466/2012 and approved by the Research Ethics Committee at the State University of Campinas (UNICAMP) (CAAE: 55933516.3.0000.5404). The study protocol was previously published in *BMJ Open* (BMJ Open, 2018; doi:10.1136/bmjopen-2017-019505) [16]. All participants provided written informed consent prior to study enrollment.

### 2.11. Allocation

The allocation concealment mechanism was carried out through opaque envelopes. The list was kept in the possession of an independent researcher, with no involvement in the participant recruitment process.

### 2.12. Blinding

Due to the nature of the interventions, blinding of the participants and researchers involved in the care was not possible, only blinding of the data analysts.

### 2.13. Data Management

Each participant had a paper file to record the baseline, daily assessments, and outcomes. The data were recorded in electronic files throughout the study to ensure confidentiality, and to preserve the participants′ identities, each individual was identified by a unique code.

### 2.14. Statistical Methods

To describe the sample profile according to the variables under study, frequency tables of categorical variables were created with absolute frequency values (*n*) and percentages (%), and descriptive measures (mean, SD, minimum, median, and maximum) were calculated for numerical variables. The normality of quantitative data was assessed using the Shapiro–Wilk test. For variables that did not meet the assumption of normality, comparisons between groups were performed using the nonparametric Mann–Whitney test, while normally distributed variables were analyzed using Student′s *t*‐test. Categorical variables were compared using the chi‐square test or Fisher′s exact test, as appropriate. Temporal analysis was conducted using repeated measures analysis of variance (ANOVA). Factors associated with the number of days to healing were evaluated using simple and multiple Poisson regression with stepwise selection. The significance level was set at 5%, and all analyses were conducted blinded to group allocation to ensure objectivity.

### 2.15. Data Sharing

Individual deidentified participant data, along with the data dictionary, statistical code, and other related materials, are available upon reasonable request from the corresponding author. Access to the data may be granted following approval by the research ethics committee and the signing of a data sharing agreement.

## 3. Results

### 3.1. Participant Flow

Between October 2022 and November 2024, a total of 62 patients were screened for eligibility in the study. Among these, three did not meet the inclusion criteria, and 15 declined participation. Consequently, 44 patients (70%) provided informed consent and were randomized, with 22 allocated to the TG, which received treatment with the *A. chica* mucoadhesive gel, and 22 to the CG, treated with low‐level laser therapy.

During the study, two participants from the TG were withdrawn: one due to the need for intensive care unit (ICU) admission resulting from oncological treatment complications, and another due to death secondary to graft failure, leaving 20 participants who completed the protocol. In the CG, three participants discontinued the intervention: one due to ICU admission following oncological complications, one due to death resulting from severe infection associated with intense immunosuppression, and one due to hospital discharge before completing the OM treatment protocol.

**Figure 1:**
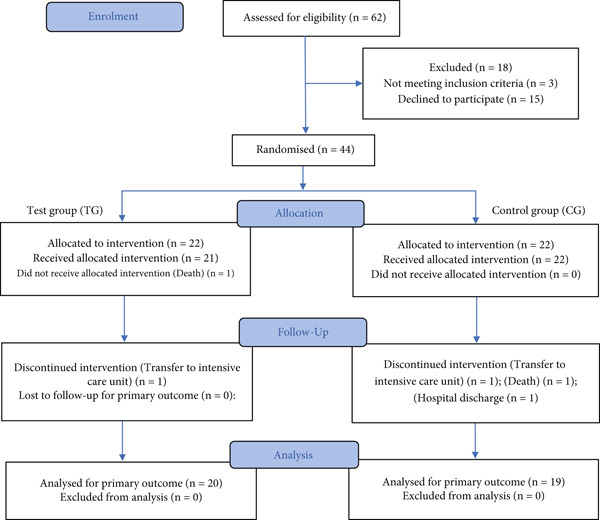
CONSORT 2025 flow diagram showing the progress through the phases of the randomized trial with two groups (test group [TG] and control group [CG]): enrolment, intervention allocation, follow‐up, and data analysis.

As illustrated in the CONSORT flow diagram (Figure 1), 20 participants in the TG and 19 in the CG completed the study protocol. In accordance with CONSORT recommendations, it is important to note that the trial has not been terminated or concluded; the data presented correspond to an interim analysis from one participating center (Hospital de Clínicas, State University of Campinas [UNICAMP]). The clinical trial remains ongoing at both participating study centers to achieve the final planned sample size.

The average age of the participants in the TG was 49.2 years, with a predominance of 11 (55%) males and 9 (45%) females. In the CG, the average age was 45.5 years, with 10 (42.6%) male participants and 9 (47.4%) female participants. In the TG, cases of leukemia, lymphoma, and myeloma were identified, with a predominance of multiple myeloma. Most participants in this group underwent autologous transplant, while a smaller proportion received an allogeneic graft. In the CG, the diagnoses also included leukemia, lymphoma, and multiple myeloma, with the latter being the most frequent. Autologous transplant was more common in this group, followed by allogeneic transplant. There was no statistical difference for any of these parameters, as shown in Table 2.

**Table 2:** Demographic and clinical characteristics of the intervention (IG) and control (CG) groups.

**Characteristics**	**IG**	**CG**	**p** **value**
Sample size	*n* = 20	*n* = 19	
Age (mean ± SD)	49.2 ± 15.1	45.5 ± 14.9	0.4789
Male gender (%)	11 (55%)	10 (52.6%)	0.9337
Female gender (%)	9 (45%)	9 (47.4%)	
Oncohematological disease (%)			
Acute lymphoid leukemia	3 (15%)	2 (10.5%)	
Acute myeloid leukemia	1 (5%)	5 (6.3%)	
Chronic myeloid leukemia	1 (5%)	1 (5.3%)	
Non‐Hodgkin lymphoma	4 (20%)	1 (5.3%)	
Hodgkin lymphoma	1 (5%)	2 (10.5%)	
Dysplastic myeloma	1 (5%)	1 (5.3%)	
Multiple myeloma	9 (45%)	7 (36.8%)	
Type of transplant (%)			
Autologous *n* = (*%*)	12 (60%)	11 (57.9%)	0.7738
Allogeneic *n* = (*%*)	8 (40%)	8 (42.1%)	

As expected, some participants in the intervention group reported a mild burning sensation upon application of the gel to the lesion; however, this effect did not compromise the treatment efficacy or the acceptability of the intervention.

### 3.2. Outcome

#### 3.2.1. Primary Outcome

OM was monitored through serial clinical images from the first day of study inclusion until complete lesion healing (Figure 2). The mean healing time was significantly shorter in the TG compared to the CG. In the TG, the mean healing time was 5.2 ± 1.5 days (95% CI: 4.45–5.95; median: 5.0; range: 2–7), whereas in the CG it was 11.4 ± 6.5 days (95% CI: 7.94–14.86; median: 11.0; range: 5–33), with a *p* value of < 0.0001. These results indicate a faster resolution of lesions in patients treated with the *A. chica* gel.

Figure 2Serial clinical images of oral mucositis in a patient randomized to the test group (TG) after hematopoietic stem cell transplantation. (a) Baseline (day of randomization). (b) Day 4 of treatment with *Arrabidaea chica* mucoadhesive gel. (c) Day 6, showing complete healing of the lesion.

**Figure:**
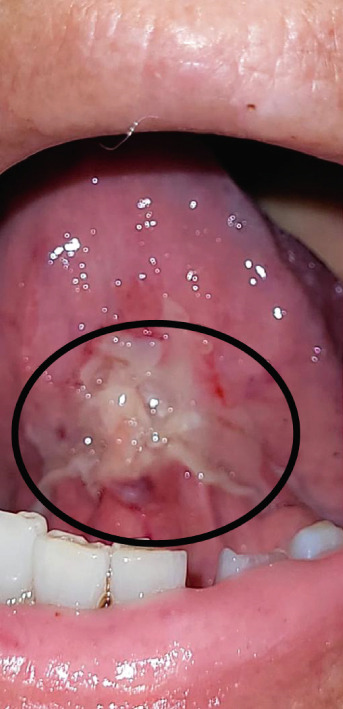
(a)

**Figure:**
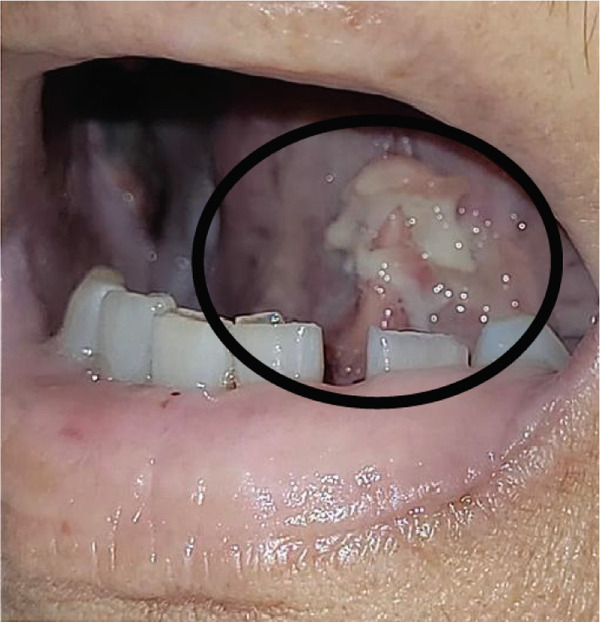
(b)

**Figure:**
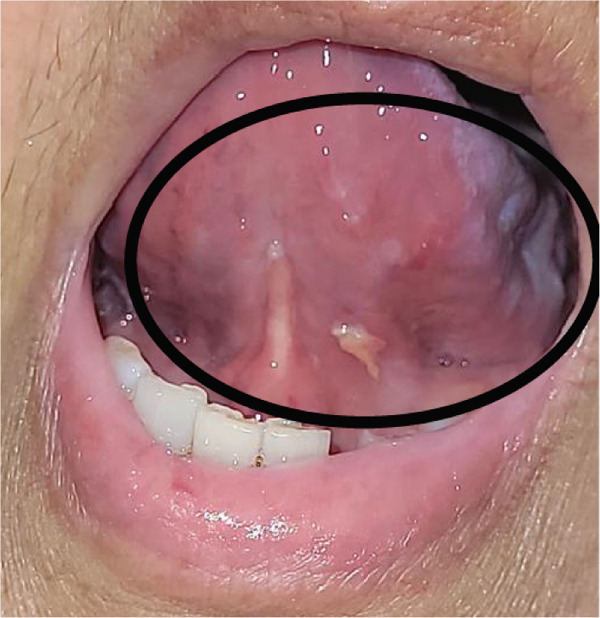
(c)

#### 3.2.2. Secondary Outcome

Pain intensity associated with OM was assessed using the VAS. Baseline pain scores differed between groups, with a mean of 6.1 ± 2.8 in the TG and 4.3 ± 2.7 in the CG, indicating a potential baseline imbalance. To account for this difference, an analysis of covariance (ANCOVA) was performed using baseline VAS scores as a covariate. After adjustment, both groups showed a substantial reduction in pain by the end of the study, with adjusted mean VAS scores of 0.1 ± 0.3 (95% CI: 0.0–0.2) for the TG and 0.0 ± 0.0 (95% CI: 0.0–0.0) for the CG, confirming the effectiveness of the intervention.

Health‐related quality of life, evaluated by the EQ‐5D questionnaire, also improved over time in both groups. Adjusted for baseline differences, the mean EQ‐5D scores increased from 0.8 ± 0.2 (95% CI: 0.7–0.9) to 0.9 ± 0.1 (95% CI: 0.8–0.9) in the TG and from 0.7 ± 0.3 (95% CI: 0.6–0.9) to 1.0 ± 0.1 (95% CI: 0.9–1.0) in the CG. Similarly, participants′ perception of overall health status improved, with adjusted scores rising from 68.5 ± 17.2 (95% CI: 58.9–78.1) to 86.3 ± 16.5 (95% CI: 77.3–95.3) in the TG and from 68.8 ± 20.0 (95% CI: 57.7–79.9) to 88.8 ± 12.0 (95% CI: 81.3–96.3) in the CG, reflecting enhanced perceived health in both groups.

## 4. Discussion

In this study, despite prophylaxis with low‐intensity laser, 50% of participants developed OM of Grades 1–4, according to the WHO classification. These findings align with the literature, which reports incidences ranging from 30% to 75%, depending on the characteristics of the transplant and the patients. The high observed rate emphasizes the need for further research to improve prophylactic and treatment strategies, suggesting that laser therapy may not be fully effective in preventing and reducing the severity of OM [17–19]. However, these data refer to patients who did not undergo prophylactic treatment for OM.

When evaluating the demographic and clinical characteristics of both groups, we observed an equilibrium between them. This similarity suggests that the comparison of results is valid, minimizing the potential for bias related to age and sex. This parameter aligns with data found in the scientific literature, which indicates that a balanced sex distribution is essential for the validity of results in clinical studies [18, 20].

Regarding the type of graft, the most predominant in both groups, IG and CG, was autologous, with 61.1% and 56.3% of cases, respectively. However, these data contrast with the observations of Wysocka‐Słowik et al. [21], who identified a higher incidence of OM in patients undergoing allogeneic HSCT.

Regarding the development period of OM after transplantation, no statistical difference was observed between the groups, occurring in the IG at an average of 7.8 ± 4.2 days and in the CG at 7.9 ± 4.3 days after stem cell infusion. Although prophylactic treatment was administered to all participants in this study, the time of lesion onset corroborates the literature, which indicates the first signs of OM in untreated patients between the first and second week after stem cell infusion [21, 22]. These findings reinforce the idea that the use of laser may not be as effective in preventing OM.

The most prevalent degree of OM in the IG was Grade 3, affecting 44.4% of the population, followed by Grades 2 and 4, each at 22.2%. These data corroborate a 2021 study that assessed the severity of OM according to the WHO classification in patients undergoing oncohematological treatment, finding a significant frequency of severe cases (Grades 2–4) between the first and second weeks post‐BMT in the analyzed population [21]. In the CG, 50% of participants presented Grade 1 OM, followed by 31% with Grade 2. These findings highlight the need for an individualized approach to OM management, considering not only prophylactic methods but also the continuous evaluation of the effectiveness of interventions and the clinical support provided. Understanding the reasons behind the differences in OM grades between the groups could lead to more effective treatment strategies and improve the quality of life for patients undergoing oncological treatment.

Regarding pain associated with MO, the initial average pain in the IG was 6.1 ± 2.8, which decreased to 0.1 ± 0.2 by the end of the study. In the CG, the starting pain score was 4.3 ± 2.7, dropping to 0.0 ± 0.0 by the study′s conclusion. Reports indicated that the IG experienced greater pain perception than the CG at the study′s onset, but both groups completed the protocol without reporting pain at the end. The study results suggest that although prophylactic treatment did not reduce lesion severity, pain intensity appeared to decrease, as reported by the participants. In accordance with the MASCC/ISOO guidelines, low‐intensity laser use in both prophylaxis and treatment was effective in pain reduction. However, no reduction in lesion severity was observed, as noted by Lalla et al. [3].

The results indicate that the healing time in the IG, despite more severe lesions, is significantly faster than in the CG, being 2.2 times quicker. This suggests that the use of the gel has a considerable impact on accelerating the healing process of OM. This improvement not only positively affects the patients′ quality of life but also has significant economic implications and may potentially prevent interruptions in oncological treatment due to OM‐related complications.

Studies highlight the complexity of the oral cavity, emphasizing the need to develop strategies that improve topical administration, with mucoadhesive formulations emerging as a promising alternative. The oral cavity consists of a multifunctional system composed of the epithelium and underlying tissue, whose anatomical differences influence drug permeability and formulation retention [23]. In this context, the mucoadhesive gel of *A. chica* demonstrates healing properties in a short period, in addition to promoting effective adhesion to the oral mucosa, allowing convenient and efficient administration. Furthermore, this route avoids first‐pass metabolism, increasing bioavailability, providing rapid action, reducing the incidence of adverse effects, and ensuring high patient adherence [24].

Regarding safety, no adverse reactions associated with the use of the mucoadhesive gel were reported. This absence of side effects supports previous studies showing that *A. chica* extracts did not cause significant clinical changes [12, 14]. These results reinforce the feasibility of the mucoadhesive gel as a safe option for managing OM.

The mucoadhesive gel provides uniform coverage of the oral and esophageal mucosa, ensuring better adherence to the tissue and effectiveness in treatment. The at‐home application offers convenience, enhances therapeutic adherence, and serves as a cost‐effective alternative, benefiting both patients and the healthcare system. In contrast, low‐intensity laser therapy requires investments in equipment, professional training, and an appropriate environment, as well as presenting challenges in precise positioning, especially in patients who have difficulty keeping their mouth still, which may compromise its effectiveness.

The analysis of the baseline scores from the EQ‐5D questionnaire reveals that there was no statistically significant difference between the groups. This suggests that the initial differences in perceived quality of life between the groups are minimal and may not have clinical relevance. The final scores also did not show a statistically significant difference. Furthermore, these data reflect the patients′ health perception at the time of the assessment, highlighting the subjectivity of the responses and the importance of considering the individual context of each participant when interpreting the results.

We conclude that the mucoadhesive gel of *A. chica* promotes OM healing in a significantly shorter time than low‐intensity laser, even in more severe lesions, achieving complete recovery 2.2 times faster. In addition to efficacy, the gel offers an accessible and easy‐to‐apply alternative, requiring minimal infrastructure. The absence of adverse events suggests a favorable safety profile, although studies with larger sample sizes and in different clinical contexts are necessary to validate its long‐term safety. The findings are promising and open new perspectives for the development of innovative interventions in OM healing, representing a significant advancement in the treatment of this debilitating condition.

## 5. Limitations

Although the results reached statistical significance, the relatively small sample size may have limited the ability to detect differences in some secondary outcomes and subgroup analyses, as well as restricted the generalizability of the findings to other populations.

## Ethics Statement

This study was approved by the Ethics Committee of the University of Campinas (UNICAMP). All participants signed the informed consent form.

## Disclosure

The full trial protocol and other relevant documents, including additional on harms, are available in the publication “*Arrabidaea chica* for Oral Mucositis in Patients With Head and Neck Cancer: A Protocol of a Randomised Clinical Trial” (BMJ Open, 2018; doi:10.1136/bmjopen-2017-019505).

## Conflicts of Interest

The authors declare no conflicts of interest.

## Funding

The study is supported by Fundação de Amparo à Pesquisa do Estado de São Paulo, 10.13039/501100001807 (2021/01280‐3 and 2018/20252‐8), and Conselho Nacional de Desenvolvimento Científico e Tecnológico, 10.13039/501100003593 (300724/2022‐9).

## Data Availability

The data that support the findings of this study are available on request from the corresponding author. The data are not publicly available due to privacy or ethical restrictions.
